# Supplementary material for: Guideline-based bismuth quadruple therapy for helicobacter pylori infection in China: A systematic review and network meta-analysis
Source: PLoS One. 2025 Feb 20;20(2):e0318937. doi: 10.1371/journal.pone.0318937 (PMC11841909; doi:10.1371/journal.pone.0318937)
Supplement: S1 Table — The table of search strategies. (DOCX) [file pone.0318937.s001.docx]

**Supplementary Table1. Search strategy**

| **Database** | **Search strategy** |
| --- | --- |
| PubMed | (("helicobacter pylori"[MeSH Terms] OR "helicobacter pylori"[Title/Abstract] OR "helicobacter pylon"[Title/Abstract] OR "helicobactor pylori"[Title/Abstract]) AND ("random"[Title/Abstract] OR "randomized"[Title/Abstract] OR "randomization"[Title/Abstract] OR "randomized"[Title/Abstract] OR "randomised"[Title/Abstract]) AND "China"[Text Word] AND ("humans"[MeSH Terms] AND "english"[Language])) AND ((humans[Filter]) AND (english[Filter])) |
| the Cochrane Library | (helicobacter pylori OR helicobacter pylon OR helicobactor pylori) in Title Abstract Keyword AND (Random*) in All Text |
| Web of Science | (TS=(helicobacter pylori or helicobacter pylon)) AND AB=(randomize or random or randomization or random or randomized) |
| SinoMed | ("幽门螺旋杆菌"[标题] OR "幽门螺杆菌"[标题] OR "幽门螺旋菌"[标题] OR "helicobacter pylori"[标题] OR "helicobacter pylon"[标题] OR "helicobactor pylori"[标题]) AND ("随机"[全部字段]) NOT ("动物"[标题] OR "鼠"[标题] OR "兔"[标题] OR "猴"[标题] OR "狗"[标题]) |
| CNKI | (SU='幽门螺旋杆菌'+'幽门螺杆菌'+'幽门螺旋菌'+'helicobacter pylori'+'helicobacter pylon'+'helicobactor pylori') and AB='随机' NOT (AB='鼠'+'兔'+'动物') |
| Wanfang | 主题:(幽门螺旋杆菌 or 幽门螺杆菌 or 幽门螺旋菌 or helicobacter pylori or helicobacter pylon or helicobactor pylori) and 摘要:(随机) not （摘要:动物 or 鼠 or 兔） |
| VIP | (M=幽门螺杆菌 OR M=幽门螺旋杆菌 OR M=幽门螺旋菌 OR M=helicobacter pylon OR M=helicobacter pylori) AND (U=随机) |
