# Supplementary material for: Guideline-based bismuth quadruple therapy for helicobacter pylori infection in China: A systematic review and network meta-analysis
Source: PLoS One. 2025 Feb 20;20(2):e0318937. doi: 10.1371/journal.pone.0318937 (PMC11841909; doi:10.1371/journal.pone.0318937)
Supplement: Attachment — Submitted filename: Response to Reviewers.docx [file pone.0318937.s005.docx]

**Response to Reviewers**

Title: Guideline-based for Helicobacter pylori infection in China: A Systematic Review and Network Meta-analysis of Bismuth Quadruple Therapy

Authors: Jiali Wei, Zehao Zheng, Xin Wang, Boyi Jia, Mingyao Sun, Jiayi Wang, Qin Wan, Mei Han, Yue Qiu

Manuscript lD: PONE-D-24-31187

**Dear Editors,**

Thank you for giving us the opportunity to submit a revised draft of the manuscript titled "Guideline-based for Helicobacter pylori infection in China: A Systematic Review and Network Meta-analysis of Bismuth Quadruple Therapy" for publication in *PLOS ONE*. **We deeply appreciate the time and effort that you and the reviewers** dedicated to providing feedback on our manuscript, and we are grateful for the insightful comments and valuable suggestions that have significantly improved our paper.

**We have carefully considered all the feedback provided and have made revisions accordingly.** The changes made to the manuscript are highlighted within the marked-up copy. We believe that these revisions have strengthened the clarity and quality of the manuscript, and **we are confident that it is now in a much better form for publication.**

As requested, we are submitting the following materials:

1. A rebuttal letter responding to each point raised by the academic editor and reviewer(s).
2. A marked-up copy of the manuscript, with all changes highlighted.
3. An unmarked version of the revised manuscript, with no tracked changes.

Below, we have outlined the specific modifications made in response to the reviewers' comments:

1. Our manuscript is now fully complies with PLOS ONE's style requirements.
2. The [Inclusion and exclusion literature Data Sheet] is now included in the supplementary materials, as Supplementary Table S2.
3. The ORCID ID for the corresponding author is provided.
4. The following statement has been added to the manuscript: "**The funders had no role in study design, data collection and analysis, decision to publish, or preparation of the manuscript.**"
5. Captions for the Supporting Information files have been included at the end of the manuscript.
6. A copy of **Table 1** has been included on **page 10.**

Thank you for your careful review and for the valuable suggestions provided by **Reviewer 1**. In response to the reviewer's comments, I have made the following revisions and clarifications:

1. Regarding the mention of "the new potassium-competitive acid blocker Vonoprazan," as indicated in The Fifth Consensus Report in China, which states that Vonoprazan has a stronger acid-suppressive effect compared to existing PPIs and may further improve the H. pylori eradication rate: According to the 2022 "Chinese National Clinical Practice Guideline on Helicobacter pylori Eradication Treatment," both the bismuth-containing quadruple therapy with PPI and the bismuth-containing quadruple therapy with P-CAB are recommended as first-line and second-line treatments for H. pylori eradication (weak recommendation, low-quality evidence). **However, in the network meta-analysis conducted in this study, we selected PPIs based on higher-quality evidence from the guideline**, including esomeprazole, rabeprazole, omeprazole, lansoprazole, pantoprazole, and esomeprazole. Moreover, we based our analysis on the most commonly used combinations in clinical research. We did not retrieve any randomized controlled trials conducted in mainland China on vonoprazan, and we were unable to include it in the network analysis. If we have overlooked any relevant studies, we would appreciate it if you could recommend additional references for inclusion.
2. Regarding **the eradication rate of less than 80%, this has already been addressed in the discussion section.** Among the 130 treatment arms included in the network analysis, **27 (20.8%)** had an eradication rate of less than 80%. These arms were primarily combinations of different PPIs with clarithromycin or levofloxacin, consistent with the high resistance rates to these antibiotics reported in mainland China. This underscores the inadequacy of current H. pylori eradication methods.

Thank you for your thorough review and for the valuable comments provided by **Reviewer 2**. In response to the reviewer’s suggestions, I have made the following revisions and clarifications:

1. Regarding the mention of "The Sixth Consensus Report in China," you pointed out that it primarily emphasizes non-eradication treatment methods and diagnostic content, while the relevant H. pylori therapies are discussed in detail in **"The Fifth Consensus Report in China,"** and both consensus reports make similar recommendations for quadruple therapy. In light of this, we have updated the reference to "The Fifth Consensus Report in China."
2. Regarding the inclusion criterion “regardless of age” mentioned in the article, which seems to contradict the exclusion criterion “for children” in Figure 1, we reviewed the data extraction table and found that our study did not include children. Therefore, **we have corrected the age-related inclusion criterion.**
3. **Concerning the distinction between heterogeneity and inconsistency, we have clarified the issue.** Heterogeneity refers to differences between studies in direct comparisons, while inconsistency refers to differences in indirect comparisons within the network. Inconsistency can be viewed as a form of heterogeneity within the network structure. As the number of multi-arm studies increases, inconsistency tends to decrease. In our study, multi-arm studies accounted for over 27% (15/55), thus the inconsistency is relatively low. We reviewed published network meta-analyses and relevant textbooks, and based the description of heterogeneity (inconsistency) primarily on the P-values and I² values in the forest plots. Given that our network meta-analysis included 13 intervention measures, resulting in large forest plots, and no common control measures, this study used the league table of network meta-analysis to present the pairwise comparisons of any interventions using OR, 95% CI, and p-values.
4. **Regarding the impact of new-generation acid-suppressing drugs on bismuth quadruple regimens, we have elaborated on this point in the discussion.** "The Fifth Consensus Report in China" mentions that "Vonoprazan, a new potassium-competitive acid blocker, has a stronger acid-suppressive effect than existing PPIs, and its application is expected to further improve the H. pylori eradication rate." The 2022 "Chinese National Clinical Practice Guideline on Helicobacter pylori Eradication Treatment" states that both bismuth quadruple therapy containing PPI and bismuth quadruple therapy containing P-CAB are recommended for initial and second-line H. pylori eradication treatment (weak recommendation, low-quality evidence). Due to the limited RCTs on Vonoprazan in mainland China, it was not included in our study. We believe that future clinical studies on bismuth quadruple therapy with P-CAB and PPI should be conducted to further verify their clinical efficacy.
5. **Figure 1 has been corrected**, and relevant details regarding the inclusion and exclusion of studies in the full-text screening process have been provided.
6. **The background section has been simplified**: As per your recommendation, we have focused on the 2022 guidelines' recommendations for bismuth quadruple therapy and simplified the background accordingly.
7. **The manuscript has been proofread**: The paper has been reviewed by a native English speaker to ensure clarity and correctness of the language.
8. **The latest Chinese antibiotic resistance data has been cited in the introduction**: We have emphasized the necessity of performing subgroup analysis based on regional differences, and we also referred to the 2024 ACG Clinical Guidelines on Helicobacter pylori infection, noting the geographical and racial differences in Hp infection in the U.S. The revised text reads as follows:
   "Due to varying levels of antibiotic resistance and environmental factors, regional differences can significantly impact the effectiveness of Hp eradication therapies. For instance, the prevalence and acquisition patterns of Hp infection often correlate with socioeconomic and demographic factors, such as population density, hygiene standards, and racial composition. These differences suggest that treatment outcomes may not be uniform across regions. In China, where the geographical diversity encompasses variations in healthcare access, dietary habits, and antibiotic usage, a subgroup analysis by region becomes essential. This approach allows for a more accurate evaluation of treatment regimens, ensuring tailored strategies that address the unique characteristics and challenges of each region."
9. **Figures and tables have been revised and formatted**: Necessary adjustments and formatting have been made to ensure the logical flow and visual appeal of the figures and tables.
10. **Regarding the display of network meta-analysis results**: Since the study included 13 interventions in the network meta-analysis, it was not possible to obtain a forest plot for the comparisons of these interventions using the Bayesian model. Therefore, we used **the league table** of network meta-analysis to present the comparisons between the "row" and "column" interventions (i.e., comparisons among all interventions). Previously, due to the large size of the league table, we had placed it in the supplementary materials; however, it has now been moved to the main manuscript, with an efficacy ranking chart added to display the effect sizes and rank specific therapies.
11. **Regarding treatment duration** and H. pylori eradication rate: According to **The Fifth Consensus Report in China**, the recommended treatment duration is **7-14 days**, with C13 or C14 testing conducted **4 weeks** after treatment to assess H. pylori levels. All studies included in our data extraction reported the 4-week eradication rate as the outcome indicator.
12. **Regarding the rescue regimens**: The Fifth Consensus Report in China recommends that the choice of rescue regimens should take previous first-line treatments into account, with the principle of avoiding the reuse of prior regimens. This systematic review and network meta-analysis compare the clinical efficacy and safety of the first-line H. pylori eradication regimen recommended by Chinese guidelines. All studies included in this review focus on first-line therapies, which also serve as rescue regimens.
13. **Enhancements to the Discussion section**: I have revised the Discussion section, focusing on summarizing and interpreting the study results more clearly, and further elaborating on the clinical significance of our findings.
14. **Future clinical recommendations**: Based on the findings of this study, we have outlined the following future clinical strategies: Future strategies for Helicobacter pylori (Hp) eradication should prioritize evidence-based approaches and clinical innovations. Large-scale, multicenter studies with extended follow-up periods and comprehensive outcome measures are urgently needed to address current limitations in research and to elucidate the mechanisms underlying effective Hp treatment. Regional variations in infection prevalence and antibiotic resistance highlight the necessity of geographically tailored analyses to optimize therapeutic outcomes. Clinically, incorporating the new generation of acid-suppressing drugs into bismuth quadruple regimens offers a promising avenue for improving treatment efficacy, warranting further investigation through rigorous trials. Individualized treatment strategies, informed by local resistance patterns and patient-specific factors, should be emphasized alongside long-term monitoring to ensure sustained eradication and minimize recurrence. These combined efforts will enhance eradication rates, reduce antibiotic resistance, and provide robust evidence to refine clinical guidelines.

Thank you for your thorough review and the valuable suggestions provided by **Reviewer 3**. In response to your feedback, I have made the following revisions:

1. **Modification to the presentation of network meta-analysis results**: Since this study included 13 interventions, the Bayesian model could not generate a forest plot for pairwise comparisons. Therefore, we decided to present the results using **the league table of network meta-analysis** to compare the “column” treatments with the “row” treatments. The results of the comparisons between interventions are now expressed using OR and 95% CI. Additionally, due to the large size of the **the league table of network meta-analysis**, it was initially placed in the supplementary material; it has now been moved to the main text and supplemented with an efficacy ranking chart and area under the curve (AUC) to display the effect sizes and rank the treatments accordingly.
2. **Modification to intervention arm analysis and ranking**: Each arm in the network meta-analysis represents a combination of PPI and two antibiotics, and efficacy rankings have been provided for them. For example: E1 (Esomeprazole + B + AML + CLR); E2 (Esomeprazole + B + AML + FRZ); E3 (Esomeprazole + B + AML + LEV); L1 (Lansoprazole + B + AML + CLR); L3 (Lansoprazole + B + AML + LEV); O1 (Omeprazole + B + AML + CLR); O3 (Omeprazole + B + AML + LEV); P1 (Pantoprazole + B + AML + CLR); P3 (Pantoprazole + B + AML + LEV); R1 (Rabeprazole + B + AML + CLR); R2 (Rabeprazole + B + AML + FRZ); R3 (Rabeprazole + B + AML + LEV); R8 (Rabeprazole + B + FRZ + TE). The study found that, among the treatments for H. pylori eradication, the R8 regimen (Rabeprazole + B + FRZ + TE) performed best in terms of eradication rate, while the L1 regimen (Lansoprazole + B + AML + CLR) showed the best safety profile.

Once again, we sincerely thank you and the reviewers for your time and valuable contributions to our manuscript. Should you have any further questions or require additional revisions, please do not hesitate to contact us.

Kind regards,
Mei Han
Centre for Evidence-Based Medicine, Beijing University of Chinese Medicine, Beijing, 100029, China.
