# Supplementary material for: Guideline-based bismuth quadruple therapy for helicobacter pylori infection in China: A systematic review and network meta-analysis
Source: PLoS One. 2025 Feb 20;20(2):e0318937. doi: 10.1371/journal.pone.0318937 (PMC11841909; doi:10.1371/journal.pone.0318937)
Supplement: Attachment — Submitted filename: Response_to_Reviewers_auresp_2.docx [file pone.0318937.s006.docx]

**Response to Reviewers**

Title: Guideline-based Bismuth Quadruple Therapy for Helicobacter pylori infection in China: A Systematic Review and Network Meta-analysis

Authors: Jiali Wei, Zehao Zheng, Xin Wang, Boyi Jia, Mingyao Sun, Jiayi Wang, Qin Wan, Mei Han, Yue Qiu

Manuscript lD: PONE-D-24-31187

**Dear Editors,**

Thank you for giving us the opportunity to submit a revised draft of the manuscript titled "Guideline-based Bismuth Quadruple Therapy for Helicobacter pylori infection in China: A Systematic Review and Network Meta-analysis" for publication in *PLOS ONE*. **We deeply appreciate the time and effort that you and the reviewers** dedicated to providing feedback on our manuscript, and we are grateful for the insightful comments and valuable suggestions that have significantly improved our paper.

**We have carefully considered all the feedback provided and have made revisions accordingly.** The changes made to the manuscript are highlighted within the marked-up copy. We believe that these revisions have strengthened the clarity and quality of the manuscript, and **we are confident that it is now in a much better form for publication.**

As requested, we are submitting the following materials:

1. A rebuttal letter responding to each point raised by the academic editor and reviewer(s).
2. A marked-up copy of the manuscript, with all changes highlighted.
3. An unmarked version of the revised manuscript, with no tracked changes.

Below, we have outlined the specific modifications made in response to the comments:

1. We have updated the title to: "Guideline-Based Bismuth Quadruple Therapy for Helicobacter pylori Infection in China: A Systematic Review and Network Meta-Analysis."
2. We have thoroughly reviewed the reference list to ensure its accuracy and completeness.

Once again, we sincerely thank you for your time and valuable contributions to our manuscript. Should you have any further questions or require additional revisions, please do not hesitate to contact us.

Kind regards,
Mei Han
Centre for Evidence-Based Medicine, Beijing University of Chinese Medicine, Beijing, 100029, China.
